# Sub-Domains of Ricin’s B Subunit as Targets of Toxin Neutralizing and Non-Neutralizing Monoclonal Antibodies

**DOI:** 10.1371/journal.pone.0044317

**Published:** 2012-09-11

**Authors:** Anastasiya Yermakova, David J. Vance, Nicholas J. Mantis

**Affiliations:** 1 Division of Infectious Disease, Wadsworth Center, New York State Department of Health, Albany, New York, United States of America; 2 Department of Biomedical Sciences, University at Albany School of Public Health, Albany, New York, United States of America; Institute Pasteur, France

## Abstract

The B subunit (RTB) of ricin toxin is a galactose (Gal)−/N-acetylgalactosamine (GalNac)-specific lectin that mediates attachment, entry, and intracellular trafficking of ricin in host cells. Structurally, RTB consists of two globular domains with identical folding topologies. Domains 1 and 2 are each comprised of three homologous sub-domains (α, β, γ) that likely arose by gene duplication from a primordial carbohydrate recognition domain (CRD), although only sub-domains 1α and 2γ retain functional lectin activity. As part of our ongoing effort to generate a comprehensive B cell epitope map of ricin, we report the characterization of three new RTB-specific monoclonal antibodies (mAbs). All three mAbs, JB4, B/J F9 and C/M A2, were initially identified based on their abilities to neutralize ricin in a Vero cell cytotoxicty assay and to partially (or completely) block ricin attachment to cell surfaces. However, only JB4 proved capable of neutralizing ricin in a macrophage apoptosis assay and in imparting passive immunity to mice in a model of systemic intoxication. Using a combination of techniques, including competitive ELISAs, pepscan analysis, differential reactivity by Western blot, as well as affinity enrichment of phage displayed peptides, we tentatively localized the epitopes recognized by the non-neutralizing mAbs B/J F9 and C/M A2 to sub-domains 2α and 2β, respectively. Furthermore, we propose that the epitope recognized by JB4 is within sub-domain 2γ, adjacent to RTB’s high affinity Gal/GalNAc CRD. These data suggest that recognition of RTB’s sub-domains 1α and 2γ are critical determinants of antibody neutralizing activity and protective immunity to ricin.

## Introduction

Ricin, a natural product of the castor bean plant (*Ricinus communis*), is one of the most lethal protein toxins known [1], [2]. In its mature form, ricin consists of two distinct subunits, RTA and RTB, joined by a single disulfide bond. RTA (32 kDa) is an RNA N-glycosidase that irreversibly inactivates eukaryotic ribosomes through hydrolytic cleavage of a conserved adenosine residue within in the sarcin-ricin loop (SRL) of the 28S rRNA [3], [4]. RTB (34 kDa) is a galactose- and N-acetylgalactosamine (Gal/GalNac)-specific lectin that mediates attachment, endocytosis, and trafficking of RTA from the plasma membrane to the trans-Golgi network (TGN) and then the endoplasmic reticulum (ER)[5]. Once in the ER, RTA is transported via a process known as retro-translocation, across the ER membrane and into the cytoplasm where it refolds into its enzymatically active conformation and initiates ribosome depurination [6]. Ricin’s potency is due in large part to RTB’s ability to adhere to and be internalized by virtually all mammalian cell types [7].

**Figure 1:**
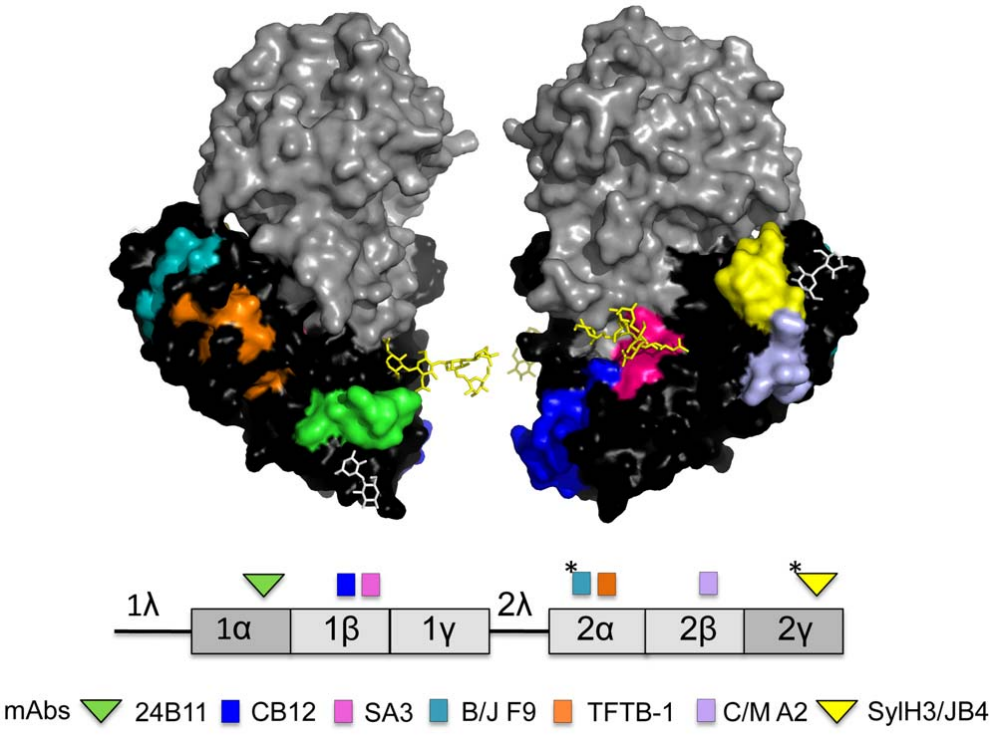
Confirmed and tentative epitopes on RTB recognized by neutralizing and non-neutralizing mAbs. (Upper panel) Surface depiction of ricin holotoxin produced by PyMOL and based on PDB file 2AAI [46]. Depicted are RTA (grey), RTB (black), ricin’s N-linked mannose side chains (yellow sticks), and lactose moieties (white sticks) situated with CRD 1α and 2γ. Specific known (and tentative) B cell epitopes recognized by RTB-specific mAbs are color-coded. (Lower panel). Linear depiction of RTB’s subdomain organization, including the leader/linker sequences (1λ, 2λ). The location of epitopes recognized by neutralizing and non-neutralizing mAbs are indicated above the figure as triangles and squares, respectively. The color of the shapes corresponds to the mAbs listed below. The asterisks indicate the putative location of mAb epitopes. See **Table 1** for a description of specific characteristics of each mAb.

Structurally, RTB consists of two globular domains with identical folding topologies (**Fig. 1**) [8]. Each of the two domains (1 and 2) is comprised of three homologous sub-domains (α, β, γ) that probably arose by gene duplication from a primordial carbohydrate recognition domain (CRD) [5], [8]. However, in the “modern” protein only the external sub-domains, 1α and 2γ, retain functional carbohydrate recognition activity [5], [9]. Sub-domain 1α (residues 17–59) is Gal-specific and is considered a “low affinity” CRD, whereas sub-domain 2γ (residues 228–262) binds both Gal and GalNac and is considered a “high affinity” CRD [10], [11], [12]. Sub-domains 1α and 2γ are separated by approximately 70 Angstroms [8].

Numerous groups, including ours, have reported that immunization of mice with RTB elicits a mixture of ricin toxin neutralizing and non-neutralizing antibodies (**Table S1**) [13], [14], [15], [16], [17], [18], [19], [20], [21], [22], [23], [24], [25], [26], [27], [28], [29], [30], [31], [32]. In an effort to identify the regions (or sub-domains) of RTB that are important in eliciting protective immunity to ricin, we recently produced and characterized a collection of two neutralizing and four non-neutralizing RTB-specific murine monoclonal antibodies (mAbs) (**Fig. 1**) [25], [32]. The epitopes recognized by the four non-neutralizing mAbs (JB11, CB12, SA3, and TFTB-1) were identified by pepscan analysis [32]. Three bound within RTB’s sub-domain 1β, while the fourth bound within sub-domain 2α (**Fig. 1**). The epitope recognized by 24B11, one of the two neutralizing mAbs we characterized, was tentatively identified through the use of a phage-displayed peptide library, as being adjacent to the Gal-specific CRD in sub-domain 1α (**Fig. 1**) [25]. The epitope recognized by SylH3, the other neutralizing mAb we characterized, has not been definitively mapped, although a preponderance of evidence would suggest that it is adjacent to the Gal/GalNac-specific CRD in sub-domain 2γ [32]. Other RTB-specific mAbs with potent toxin neutralizing activity have been reported recently, although the location of their respective epitopes was not disclosed [30].

One of the objectives of our research program is to generate a complete B cell epitope map of RTB, and to then use this information in the design of an RTB-based subunit vaccine that could be used by public health and military sectors as a countermeasure against the threat of ricin as a biological weapon [1], [33], [34]. The partial B cell epitope map we previously constructed using a limited collection of mAbs (**Fig. 1**) suggests that toxin-neutralizing mAbs bind exclusively to epitopes within sub-domains 1α and 2γ, whereas non-neutralizing mAbs cluster within the interior sub-domains (1β, 1γ, 2α, 2β). However, such a conclusion is premature without additional epitope assignments, particularly within sub-domains 1γ and 2β. Towards this end, we have screened an additional collection of ricin-specific B cell hybridomas and now describe the characterization of two new non-neutralizing and one new neutralizing RTB-specific mAbs. One of the two non-neutralizing mAbs (C/M A2) recognizes an epitope within sub-domain 2β, while the other (B/J F9) binds an epitope tentatively situated within 2α. The highly potent ricin-neutralizing mAb, JB4, recognizes an epitope within sub-domain 2γ that overlaps (or is identical to) SylH3’s binding site. These data significantly refine the current B cell epitope map of RTB and further support our hypothesis that neutralizing B cell epitopes are restricted to sub-domains 1α and 2γ, an observation that is of considerable importance in vaccine design.

## Materials and Methods

### Chemicals, Biological Reagents and Cell Lines

Ricin toxin (*Ricinus communis* agglutinin II), *Ricinus communis* agglutinin I (RCA-I), ricin toxin A subunit (RTA), and ricin toxin B subunit (RTB) were purchased from Vector Laboratories (Burlingame, CA). Ricin was dialyzed against phosphate buffered saline (PBS) at 4°C in 10,000 MW cutoff Slide-A-Lyzer dialysis cassettes (Pierce, Rockford, IL), prior to use in cytotoxicity studies. GlutaMax™, fetal calf serum and goat serum were purchased from Gibco-Invitrogen (Carlsbad, CA). Ph. D.™-12 phage display peptide library kit was purchased from New England BioLabs (Beverly, MA). A ClonaCell HY™ kit for hybridoma production was purchased from STEMCELL Technologies (Vancouver, BC, Canada). Unless noted otherwise, all other chemicals were obtained from Sigma-Aldrich (St. Louis, MO). Vero, THP-1, and the murine myeloma cell line P3X63.Ag8.653 were purchased from the American Type Culture Collection (Manassas, VA). Cell culture media were prepared by the Wadsworth Center Media Services facility. Unless otherwise noted, all cell lines and hybridomas were maintained in a humidified incubator at 37°C with 5% CO_2_.

### Mouse Strains, Animal Care and Immunizations

Female BALB/c mice approximately 8–10 weeks of age were purchased from Taconic Labs (Hudson, NY). Animals were housed under conventional, specific pathogen-free conditions and were treated in compliance with the Wadsworth Center’s Institutional Animal Care and Use Committee (IACUC) guidelines. For hybridoma production, female BALB/c mice were primed i.p. with ricin toxoid (RT, 50 µg or 2 uM per mouse in 0.4 ml PBS) on day 0, and then boosted by the same route with RT (50 µg) on days 10 and 20. RT was produced as described previously [35].

### B-cell Hybridoma Production

Four days after the second boost with RT (50 µg), mice were euthanized, and total splenocytes were fused with the myeloma cell line P3X63.Ag8.653, using polyethylene glycol (PEG) as described previously [27]. The resulting hybridomas were seeded in methylcellulose and cloned as per the instructions in the ClonaCell -HY™ hybridoma cloning manual (STEMCELL Technologies, Vancouver, BC, Canada). Hybridomas secreting antibodies of interest were expanded and cultured in either RPMI medium containing 10% fetal calf serum, oxaloacetate, pyruvate, and insulin (OPI), 8 mM GlutaMax™, and penicillin-streptomycin, or in medium A (STEMCELL Technologies) before being transitioned to CD Hybridoma, a serum-free, protein-free, antibiotic-free medium (Gibco-Invitrogen, Carlsbad, CA).

### ELISAs for Determining mAb Specificity

ELISAs were performed as previously described [27]. Briefly, Nunc Maxisorb F96 microtiter plates (ThermoFisher Scientific, Pittsburgh, PA) were coated overnight with ricin (0.1 µg/well; 15 nM), RCA–I (0.1 µg/well; 8 nM), RTA (0.1 µg/well; 31 nM), RTB (0.1 µg/well; 29 nM), BSA (0.1 µg/well; 8 nM) or peptides (1 µg/well; 3–5 µM) in PBS (pH 7.4) before being treated with hybridoma supernatants, or purified mAbs. Horseradish peroxidase (HRP)-labeled goat anti-mouse IgG-specific polyclonal antibodies (SouthernBiotech, Birmingham, AL) were used as the secondary reagent. The ELISA plates were developed using the colorimetric detection substrate 3,3′,5,5′-tetramethylbenzidine (TMB; Kirkegaard & Perry Labs, Gaithersburg, MD) and were analyzed with a SpectroMax 250 spectrophotometer, with Softmax Pro 5.2 software (Molecular Devices, Sunnyvale, CA).

### Vero Cell Cytotoxicity Assays

Vero cell cytotoxicity assays were performed as previously described [25], [26]. Briefly, Vero cells were trypsinized, adjusted to approximately 5×10^4^ cells per ml, and seeded (100 µl/well) into white 96-well plates (Corning Life Sciences, Corning, NY), and allowed to adhere overnight. Vero cells were then treated with ricin (0.01 µg/ml; 154 pM), ricin:mAb mixtures, or medium alone (negative control) for 2 hr at 37°C. The cells were washed to remove non-internalized toxin or toxin:mAb mixtures, and were then incubated for 48 hr. Cell viability was assessed using CellTiter-GLO reagent (Promega, Madison, WI). All treatments were performed in triplicate, and 100% viability was defined as the average value obtained from wells in which cells were treated with medium only.

### Passive Protection Studies

Individual mAbs (60 µg) were diluted into endotoxin-free PBS and then administered in a final volume of 0.2 ml to female BALB/c mice (ages 8–10 weeks) by i.p. injection. Twenty-four hours later, the mice were injected with ricin (2 µg; 100 µg/kg ) by the i.p. route, which is roughly equivalent to 10 LD_50_s [22], [36], [37]. Survival was monitored over a 2–6 day period. In addition, hypoglycemia was used as a surrogate marker of intoxication [26], [38]. Blood (<5 µl) was collected from the tail vein of the animals at 18–24 hr intervals. Blood glucose levels were measured with an Avia ACCU-CHEK handheld blood glucose meter (Roche, Indianapolis, IN). Mice were euthanized when they became overtly moribund and/or blood glucose levels fell below 25 mg/dl. For statistical purposes, readings at or below the meter’s limit of detection of ∼12 mg/dl were set to that value.

### Antibody Affinity Measurements and Competition Analysis

Affinity of antibodies for ricin toxin was determined by surface plasmon resonance (SPR) using a Biacore 3000 (GE Healthcare) instrument. Ricin was attached to a CM5 chip at a density of 550 to 650 RU. HEPES-buffered saline with EDTA and surfactant P20 (HBS-EP; 10 mM HEPES, pH 7.4, 150 mM NaCl, 3.4 mM EDTA, 0.005% of the surfactant P20 from GE Healthcare) was employed as the running buffer at a flow-rate of 30 µl/min. Serial dilutions of each antibody were made in HBS-EP, pH 7.4 from 600 nM to 18.75 nM, with each concentration series having at least one cycle of a buffer alone injection. Injection times were 3–4 minutes with dissociation times of 10 minutes. Regeneration of the chip surface was performed at a flow-rate of 50 µl/min by two 30 s pulses of 10 mM glycine, pH 1.5. The regeneration was followed by a 2 min stabilization period. All kinetic experiments were run a 25°C. Kinetic constants were obtained by analysis using the BIA evaluation software.

Antibody competitive binding assays by Biacore were performed with HBS-EP, pH 7.4 as the running buffer at a flow-rate of 10 μl/min. The first mAb was injected until saturation was achieved (i.e., when no significant additional rise in resonance units (RU) was observed after antibody injection.) The second competing mAb was then injected using a 2-min injection time. The amount of second mAb bound to the chip, in RU, was calculated as the RU value at 15 s after the injection minus the RU value at 15 s preceding the start of the injection. The chip surface was regenerated by short pulses with 10 mM glycine, pH 1.5, until the RU values had returned to baseline.

### Ricin Apoptosis Assays

THP-1 human monocytes (5×10^5^ cells/200 µl) were subjected to ricin (2.5 µg/ml : 38 nM) in the presence or absence of anti-RTB mAbs (20 µg/ml : 133 nM) for 5 h in an incubator in 96-well Microtest™ U-bottom tissue culture treated plates (BD) at 37°C and 5% CO_2_. After incubation, the cells were collected by centrifugation and then re-suspended in 1x binding buffer (200 μl), 100 µl of which was stained with 5 µl of Annexin V-FITC, as recommended by the manufacturer (Annexin V-FITC Apoptosis Detection Kit II from BD Pharmingen, cat# 556570). Samples were assayed for early apoptosis using a FACS Calibur (BD Biosciences). Results were reported as % cells positive for Annexin V-FITC. A minimum of 10,000 cells were analyzed per sample.

### Ricin Binding Assays

To determine if mAbs prevent ricin binding to asialofetuin (ASF), Nunc Maxisorb F96 microtiter plates (ThermoFisher Scientific) were coated with ASF (0.4 µg/well) (EY Laboratories, San Mateo, CA) in PBS (pH 7.4) for 18 hr at 4°C. Plates were washed with PBS containing Tween-20 (PBS-T; 0.05% v/v), blocked with 2% goat serum in PBS-T (0.05% v/v) and then overlayed with biotinylated ricin (50 ng/ml : 770 pM) and IgG mAbs (20 μg/ml : 133 nM) for 1 hr. The plates were washed to remove unbound toxin, labeled with avidin-HRP (0.4 μg/ml) and developed using TMB, as described above for ELISAs. To determine if mAbs bind the galactose binding pockets of ricin, plates were coated with ASF for 18 hr at 4°C. Plates were washed with PBS-T (0.05% v/v), blocked with 2% goat serum in PBS-T (0.05% v/v), overlaid with ricin (10 µg/ml) for 1 hr, then with mAbs (10 µg/ml) for 1 hr. Plates were then labeled with IgG-HRP and developed TMB, as described above for ELISAs.

### Epitope Mapping by Phage Displayed Peptide Library

C/M A2 and irrelevant IgG isotype control antibody MOPC21 were immobilized onto Falcon Polystyrene Tissue Culture Dishes at 4°C overnight (10 µg of antibody in 1.5 ml PBS : 44 nM), and then blocked with 2% PBS-BSA at 4°C for 2 hr. Phage library was diluted in 1 ml PBS to a concentration of 1.5×10^11^ pfu/ml and added to the MOPC 21 plate for 1 hour. The supernatant from the plate was transferred directly to the C/M A2 plate and allowed to bind for 1 hr. The plates were then washed with PBS-T (0.1% v/v), and a 1 ml solution of RTB (100 µg/ml : 3 µM) was added for 1 hour to elute phages from the variable site of the antibodies. The supernatant was collected and amplified in ER2738 *Escherichia coli* for 4.5 hours. The bacteria was spun down, and phage in the supernatant was precipitated by adding a 1:5 volume of 20% w/v PEG8000, 2.5 M NaCl solution and incubating at 4°C overnight. The next morning, the precipitated phages were spun down, the supernatant removed, and 1 ml of PBS was added to re-dissolve the phages. Phage titer was then determined by serial dilution and plating onto LB/Agar/IPTG/X-gal plates. This amplified phage stock from Round 1 was then diluted down to the same concentration as the starting library concentration (1.5×10^11^ pfu/ml) and served as the input to Round 2. Rounds 2 and 3 were largely the same procedure as Round 1, except that in Round 2 incubation times were 30 minutes with C/M A2, and an overnight elution with RTB. In Round 3, C/M A2 binding proceeded for 15 minutes, again with an overnight RTB elution. Unamplified Round 3 supernatant was plated on LB/Agar/IPTG/X-gal plates, and individual plaques were picked and amplified. These clonal phage stocks were then used in phage ELISAs and for DNA isolation with the Qiaprep Spin M13 Kit. B/J F9 phage display was performed as for C/M A2.

### Phage Binding ELISA

C/M A2 or B/J F9, MOPC21, and RTB were coated (RTB 10 µg/ml : 294 nM and Abs 10 µg/ml : 67 nM) in 96 well plates and then blocked with 2% PBS-BSA. Phages (at 1×10^10^ pfu/ml) were added for 1 hr, and then washed with PBS-T (0.1% v/v). Anti-M13-HRP was then added for 1 hr. The wells were again washed with PBS-T (0.1% v/v), and TMB Substrate was added. After ∼10 min, the reaction was quenched with 1 M phosphoric acid. The plate was then read on a plate reader at 450 nm.

### Peptide Inhibition ELISA

A 96 well plate was coated overnight with ricin (1 µg/ml : 15 nM) and then blocked with 2% BSA for 2 hours. A8 and C4 peptides at serially diluted concentrations were incubated with C/M A2 (5 µg/ml : 33 nM) for 1 hr. The peptide-antibody solutions were then allowed to bind to ricin for 1 hr, at which point the wells were washed with PBS-T (0.1% v/v). Goat anti-mouse IgG-HRP was then added for 1 hr, and the wells were washed again with PBS-T (0.1% v/v). TMB substrate was added for ∼10 min, and then quenched with 1 M phosphoric acid. The plate was then read on a plate reader at 450 nm.

### C4 Monomer Cytotoxicity Inhibitio

C4 peptide at 10 mM was incubated with serially diluted concentrations of C/M A2 for 2 hr at room temperature. C/M A2 alone at the same serially diluted concentrations was also incubated. Ricin toxin was then added to a final concentration of 10 ng/ml or 154 pM. After a 5 min incubation, the mixtures were applied to tissue culture 96 well plate that was seeded the night before with 1×10^4^ Vero cells per well. The toxin-antibody-peptide mixtures were allowed to incubate with the cells for 2 hours at 37°C, after which the mixture was replaced with fresh media. 48 hr later, cell viability was assessed with Cell-Titer Glo.

### Western Blots

Ricin was prepared at a concentration of 300 µg/ml or 4.6 µM in a solution of 0.05 mM EDTA, and then diluted 1:2 with 2X Laemmli sample buffer. Reduction of the disulfide bond linking RTA and RTB was achieved by the addition of β-mercaptoethanol (BME) to a final concentration of 2.3 µM. Reduced and non-reduced ricin samples were denatured by boiling for 6 min before being loaded on a 12% SDS-PAGE gel. The gel was soaked in transfer buffer (Bjerrum –Schafer –Nielsen) for 60 min, and then transferred to a nitrocellulose membrane at 10 V for 30 min using the semi dry unit (BioRad). Membrane was washed in PBS- Tween 20 (0.1%v/v) 3×5 min and blocked in 2% goat serum overnight at 4 C. Membrane was washed again in PBS-T (0.1%v/v) and primary antibodies were applied in 10 ml/membrane of blocking buffer 1–5 µg/ml (6 nM –33 nM) for 1 hr. Secondary antibody (goat-anti-mouse-HRP) was also applied for 1 hr. Membrane was washed 3×20 min in PBS-T (0.1% v/v) and developed using ECL Western blotting substrate (Pierce) as per the supplied instructions.

### Statistical Analysis and Software

Statistical analysis was carried out with GraphPad Prism 5 (GraphPad Software, San Diego, CA). The open-source molecular visualization software PyMOL (DeLano Scientific LLC, Palo Alto, CA) was used for epitope modeling.

## Results

### Identification and Characterization of Additional RTB-specific mAbs with Ricin Neutralizing Activity

**Figure 2:**
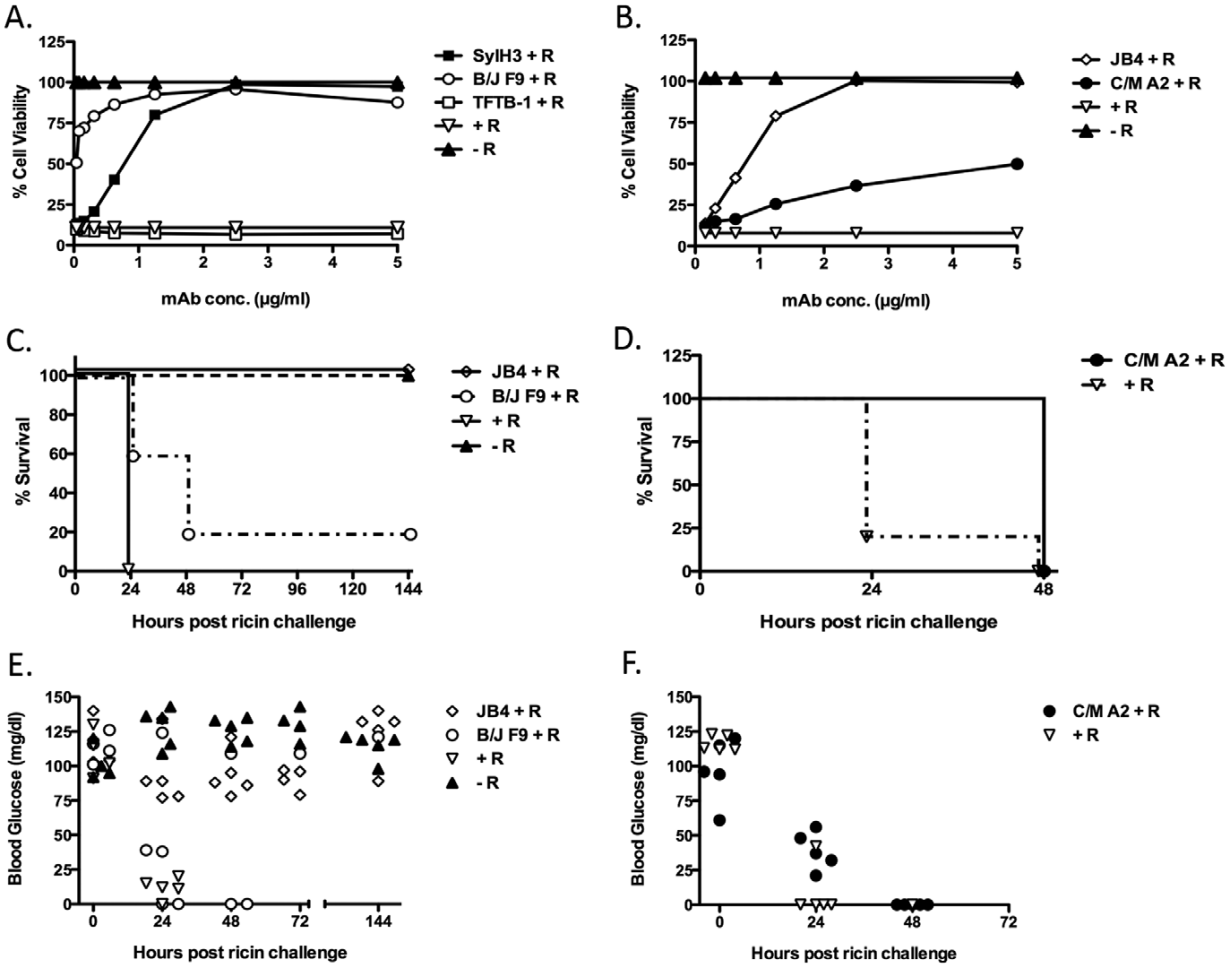
*In vitro* and *in vivo* neutralizing activity of JB4, C/M A2 and B/J F9. (Panels A,B) mAbs JB4, C/M A2, B/J F9, SylH3, or TFTB-1 were assessed for their capacity to protect Vero cells from the cytotoxic effects of ricin. TFTB-1 is a non-neutralizing RTB-specific IgG_1_ mAb that binds a linear epitope within sub-domain 2α (residues 169–184). TFTB-1 served as a negative control for these studies. Ricin (10 ng/ml; 154 pM) was incubated for 1 hr with each mAb at the indicated concentrations (starting at 5 µg/ml; 33 nM), and then applied in triplicate to Vero cells grown in 96-well microtiter plates. Cell viability was assessed 48 hr later. Each symbol (with SEM) represents the average of at least three replicate wells. This experiment was repeated at least 3 times. (**Panels** **C, D**) Passive protection studies in which groups of BALB/c mice (n = 5 per group) were injected (i.p.) with the indicated mAbs (60 μg mAb per animal) and then challenged 24 hr later by the i.p. route with 10xLD_50_s of ricin (2 µg per animal). Shown (y-axis) is percent survival as a function of time (x-axis). Mice that were challenged with ricin, but not mAb-treated were considered positive controls for this experiment and indicated by the (+ R) symbol. Mice that were sham challenged were considered negative controls and are indicated by the (− R) symbol. P value for B/J F9 vs. positive control  = 0.0495, p value or C/M A2 vs. positive control  = 0.0143. (**Panels E, F**) Blood glucose levels in the individual mice treated with indicated mAb. Blood glucose levels were determined at time 0 and then at 24 h intervals there after following ricin challenge. Each symbol represents an individual mouse. Normal blood glucose levels were considered to be >80 mg/dl. Mice with blood glucose levels <25 mg/dl were euthanized. This experiment was performed 2 times. Abbreviations: R, ricin.

We screened a collection of ∼1000 hybridomas produced from RT immunized BALB/c mice in an effort to identify additional RTB-specific mAbs capable of neutralizing ricin toxin. The screen yielded three mAbs of interest, JB4, C/M A2 and B/J F9. The remaining hybridomas were not further characterized as they did not secrete antibodies that reacted with RTB or they failed to neutralize ricin. In a Vero cell cytotoxicity assay, JB4 and B/J F9 each demonstrated a dose-dependent capacity to neutralize ricin. With IC_50_s of approximately 0.80 (5.3 nM) and 0.04 µg/ml (0.27 nM) respectively, JB4 and B/J F9 are as effective (if not slightly more effective) than the previously described mAb SylH3 at inactivating ricin in a Vero cell-based assay (**Table 1**; **Fig. 2 A, B**). C/M A2 also neutralized ricin in a dose-dependent manner, but less effectively than either JB4 or B/J F9 (**Table 1**; **Fig. 2B**). SPR analysis using a Biacore instrument revealed that JB4, C/M A2 and B/J F9 each bound ricin holotoxin with affinities roughly equal to (or slightly greater than) other previously described RTB-specific neutralizing mAbs, including SylH3 and 24B11 (**Table 1**). We also used Biacore to perform mAb competition studies, as done previously by our laboratory [27], [39]. We found that when tested in series JB4, C/M A2 and B/J F9 were largely unaffected in their abilities to bind ricin holotoxin when the Biacore chips were first saturated with one or both of the other mAbs (data not shown). On the other hand, the binding of JB4 to ricin was virtually eliminated when the toxin was first exposed to SylH3, indicating that the two mAbs recognize the same or a similar epitope on RTB (**Table S2**).

**Table 1:** Properties of RTB-specific mAbs produced in this study.

mAb	Isotype	SubD.	Epitope	K_D_ [M]	Vero cells-IC_50_ µg/ml (nM)	THP-1 cells- apoptosis	Passive protection
24B11^a^	IgG_1_	1α	38–43	4.2×10^−9^	0.60 (4)	+	+
SylH3^b^	IgG_1_	n.d.	n.d	3.38×10^−9^	0.75 (5)	+	+
JB4	IgG_1_	n.d.	n.d.	2.01×10^−10^	0.80 (5.3)	+	+
B/J F9	IgG_1_	n.d.	n.d	6.42×10^−10^	0.04 (0.27)	–	–
C/M A2	IgG_1_	2β	194–198	2.2×10^−9^	5 (33)	–	–
TFTB-1^a^	IgG_1_	2α	169–184	5.63×10^−9^	–	–	–

a , as reported by McGuinness and Mantis [25];

b , as reported by Yermakova and Mantis [32].

**Figure 3:**
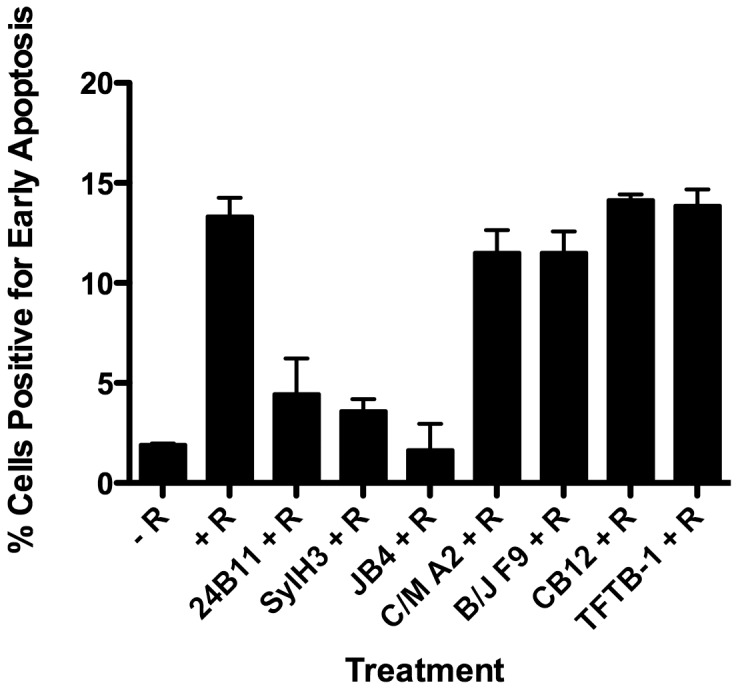
*In vitro* ricin-induced apoptosis of THP-1 cells. mAbs were assessed for their capacity to protect THP-1 human monocytes from apoptosis in the presence of ricin. Ricin (2.5 µg/ml; 38 nM) was incubated with indicated mAbs (20 µg/ml; 133 nM) for 30 min, and then added to THP-1 cells (5×10^5^/200 µl). The cell-mAb mixtures were incubated for 5 h at 37°C, then stained with FITC-Annexin V to assess apoptosis, as described in Materials and Methods. As controls, cells were treated (+ R) or not (− R) with ricin. Each bar represents the average of two replicates plotted as the mean with SEM. Abbreviations: R, ricin. This experiment was repeated 4 times.

We recently demonstrated that THP-1 cells are highly susceptible to ricin intoxication and that ricin uptake into these cells is mediated in part by the mannose receptor (MR; CD206) [40]. Therefore, we also examined the ability of JB4, C/M A2 and B/J F9 to protect THP-1 cells from ricin-induced apoptosis THP-1 cells were incubated with ricin or ricin-mAb complexes for 5 hours at 37°C before being subjected to Annexin V-FITC staining and flow cytometry. Annexin V staining was ∼7 times greater on ricin-only treated cells as compared to control, untreated cells, thereby confirming ricin’s capacity to initiate apoptosis in THP-1 cells (**Fig. 3**). Incubation of ricin with the previously described RTB-specific neutralizing mAbs, SylH3 and 24B11, blocked ricin-induced apoptosis to a large degree, whereas the addition of the non-neutralizing mAbs TFTB-1 and CB12 had no effect on toxin-induced cell killing (**Fig. 3**). The new mAbs were then tested in turn: JB4 performed as effectively (if not slightly better than) SylH3 and 24B11 in neutralizing ricin in this assay, further demonstrating that is a potent toxin neutralizing mAb. In contrast, neither C/M A2 nor B/J F9 protected THP-1 cells from ricin-induced apoptosis (**Fig. 3**). While this outcome was not surprising in the case of C/M A2, which was relatively ineffective at neutralizing ricin in the Vero cell assay (described above), it was unexpected in the case of B/J F9, which was as effective as JB4 at neutralizing ricin in the Vero cell assay (**Table 1**). Based on the results of THP-1 cell-killing assays, we predicted that JB4, but not B/J F9 and C/M A2, would be capable of neutralizing ricin *in vivo*.

Therefore, we compared JB4, B/J F9 and C/M A2 for their ability to passively protect mice against a systemic ricin challenge. Groups of BALB/c mice were administered individual mAbs (60 µg) by i.p. injection, and then challenged by the same route with 10xLD_50_s of ricin toxin 24 hr later. Immunity to ricin was assessed by two means: onset of hypoglycemia, a well-established surrogate marker of ricin toxicosis [38] and mean time to death. JB4 fully protected mice against ricin-induced death (**Fig. 2C**), although the animals did experience an acute reduction in blood glucose at 24–72 hr post challenge (**Fig. 2E**). In this respect, JB4 is comparable to other previously described RTB-specific mAbs, including SylH3 [32]. B/J F9 and C/M A2, on the other hand, were not sufficient to protect mice against ricin intoxication. Only a single mouse (1/5) administered B/J F9 survived ricin challenge and no mice (0/5) treated with C/M A2 survived toxin exposure (**Fig. 2C, D**). Moreover, following ricin challenge, mice treated with B/J F9 or C/M A2 experienced declines in blood glucose levels that were indistinguishable from the control, toxin-only treated animals (**Fig. 2E, F**). The mAbs did counteract the effects of ricin to some degree *in vivo*, as evidenced by the fact that B/J F9 (p<0.05) and C/M A2 (p<0.05) each delayed time to death, as compared to control, toxin-only treated animals. These data demonstrate that among the three new mAbs identified in this study only JB4 was capable of protecting mice against ricin intoxication.

### Differential Capacities of JB4, B/J F9 and C/MA2 to Block Ricin Attachment to Cell Surface Receptors

**Figure 4:**
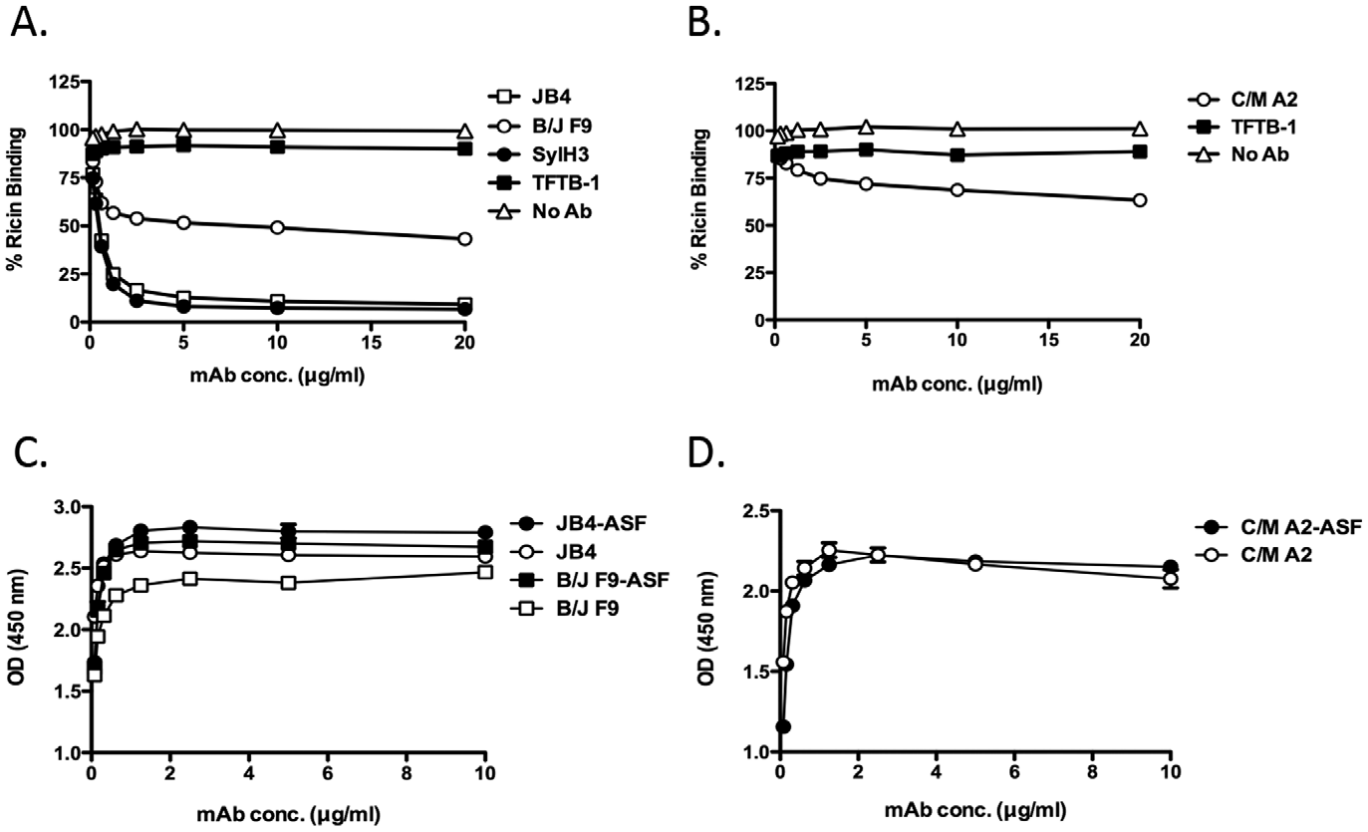
Assessing inhibition of ricin binding by individual mAbs. (Panels A, B) Biotin-labeled ricin (50 ng/ml: 770 pM) was mixed with indicated mAbs (20 µg/ml; 133 nM) and then applied to 96-well microtiter plates coated with ASF (4 µg/ml), as described in Materials and Methods. The percent binding of biotin-ricin to ASF was then detected using a standard ELISA protocol in which plates were treated with avidin-HRP and TMB substrate. Each symbol represents the average of at least three replicate wells. (**Panels C, D**) Differential reactivity of indicated mAbs with ricin or ricin-receptor complexes. Ninety-six well microtiter plates coated with ricin (open shapes) or ricin-ASF (closed shapes) were probed with indicated mAbs (10 µg/ml: 66.7 nM) JB4 and B/J F9 (**C**), and C/M A2 (**D**). This experiment was repeated at least 2 times.

It is postulated that RTB-specific mAbs neutralize ricin primarily by interfering with toxin attachment to cell surface glycoprotein and glycolipid receptors [41]. We used a quantitative solid-phase binding assay to assess the capacities of JB4, B/J F9 and C/M A2 to block ricin attachment to terminal Gal/GalNAc residues (see Materials and Methods). Biotin-labeled ricin (50 ng/ml) was incubated with JB4, C/M A2, B/J F9, SylH3, or TFTB-1 at a range of concentrations (0.25–20 µg/ml) and then applied to 96-well microtiter plates coated with ASF, a surrogate glycoprotein receptor [25]. As shown in **Figure 4A**, JB4 reduced ricin attachment to ASF in a dose-dependent manner, with an estimated IC_50_ of <1 µg/ml (7 nM). B/J F9 and C/M A2, on the other hand, were only moderately effective at blocking ricin attachment in this assay. Indeed, even at 20 µg/ml (133 nM), B/J F9 and C/M A2 only reduced ricin binding to ASF by ∼50% and ∼40%, respectively (**Fig. 4A, B**). Nonetheless, B/J F9 and C/M A2 were each more effective than TFTB-1, which had no detectable capacity to block ricin attachment.

To test whether JB4 interferes with ricin attachment to cell surfaces by physically binding an epitope within one (or both) of RTB’s two galactose binding pockets situated in sub-domains 1α or 2γ, we performed a modified ELISA in which 96-well microtiter plates were first coated with ASF and then secondarily coated with ricin. It has previously been argued that RTB’s two galactose binding pockets are occupied under these conditions, because ricin is bound to the solid substrate solely by virtue of its ability to bind ASF [16], [25]. Using this assay, we found JB4’s capacity to bind RTB was unaffected when the toxin was docked to ASF (**Fig. 4C**), thereby suggesting the mAb recognizes an epitope adjacent to but not within RTB’s galactose binding pockets. The binding of C/M A2 and B/J F9 to RTB were similarly unchanged when ricin was immobilized via ASF (**Fig. 4C, D**).

### Localization of the Epitopes on RTB Recognized by JB4, B/J F9 and C/M A2

**Figure 5:**
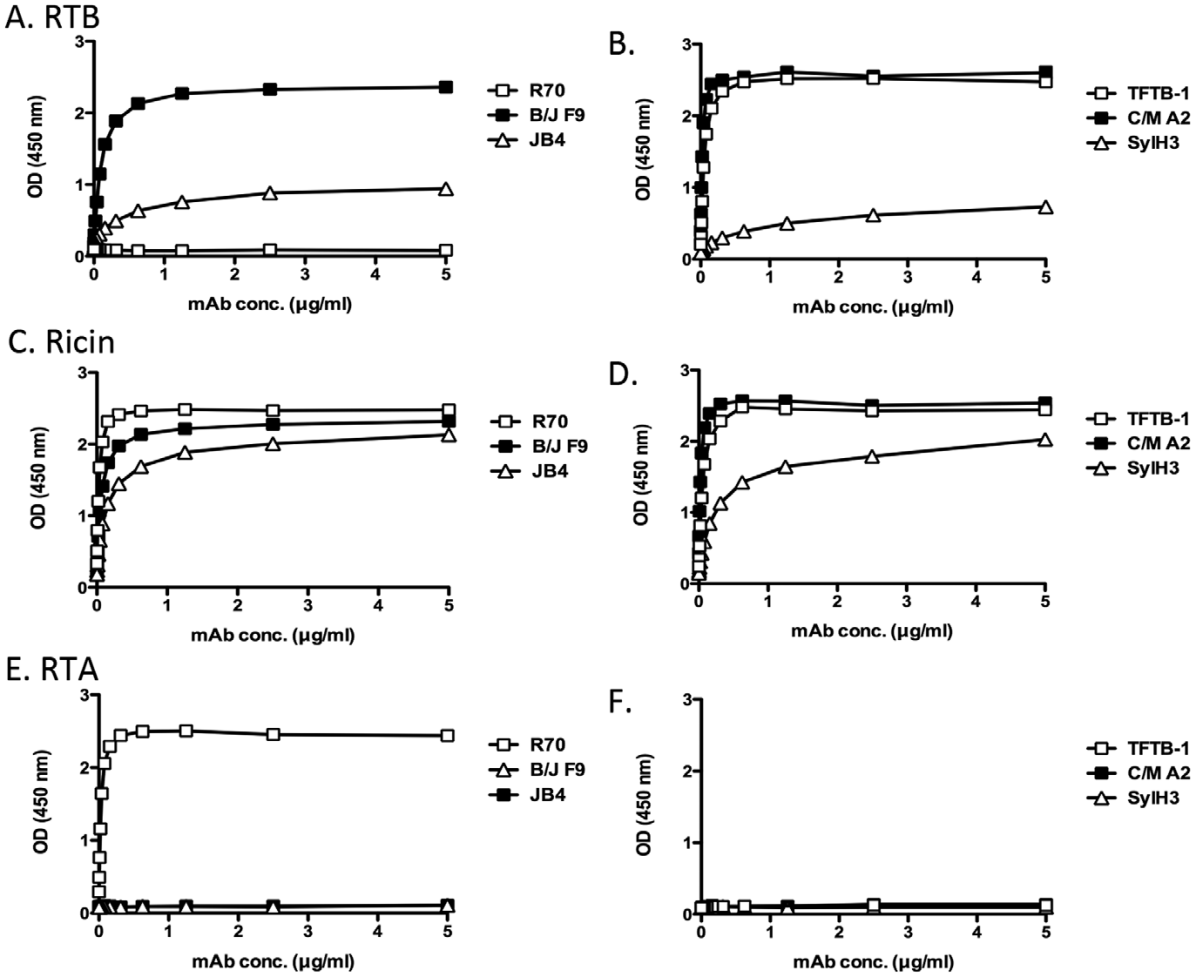
Reactivity profiles of individual mAbs with RTA, RTB and ricin holotoxin. Ninety-six well microtiter plates were coated with (**A, B**) RTB, (**C, D**) ricin holotoxin or (**E, F**) RTA and then probed with mAbs JB4, B/J F9, SylH3, TFTB-1, or R70 at indicated concentrations (starting at 5 µg/ml; 33 nM). R70 served as an RTA-specific control, while SylH3 and TFTB-1 served as RTB-specific controls. Each symbol (+/− SEM) represents the average of at least three replicate wells. The SEM may be too small to visualize in the figure. This data is representative of 3 independent experiments.

We reasoned that epitope specificity must account for the disparate capacities of JB4, B/J F9 and C/M A2 to neutralize ricin *in vitro* and *in vivo*, because all three mAbs are IgG_1_s and all three bind ricin holotoxin with similar affinities. As a first step towards identifying the regions on RTB recognized by JB4, B/J F9 and C/M A2, we compared the reactivity profiles of each mAb with ricin holotoxin and RTB. JB4 bound ricin holotoxin considerably better than RTB, suggesting that JB4 recognizes an epitope whose conformation (or accessibility) is influenced by RTB’s association with RTA (**Fig. 5 A, C**). Not surprisingly, JB4’s binding profile is strikingly similar to that of SylH3 [32]. B/J F9 and C/M A2, on the other hand, each bound RTB and ricin holotoxin equally well, demonstrating that their respective epitopes are not influenced by the proximity or association of RTB with RTA (**Fig. 4A–D**). As expected, none of the mAbs bound purified RTA to any appreciable degree (**Fig. 4** **E,F**).

**Figure 6:**
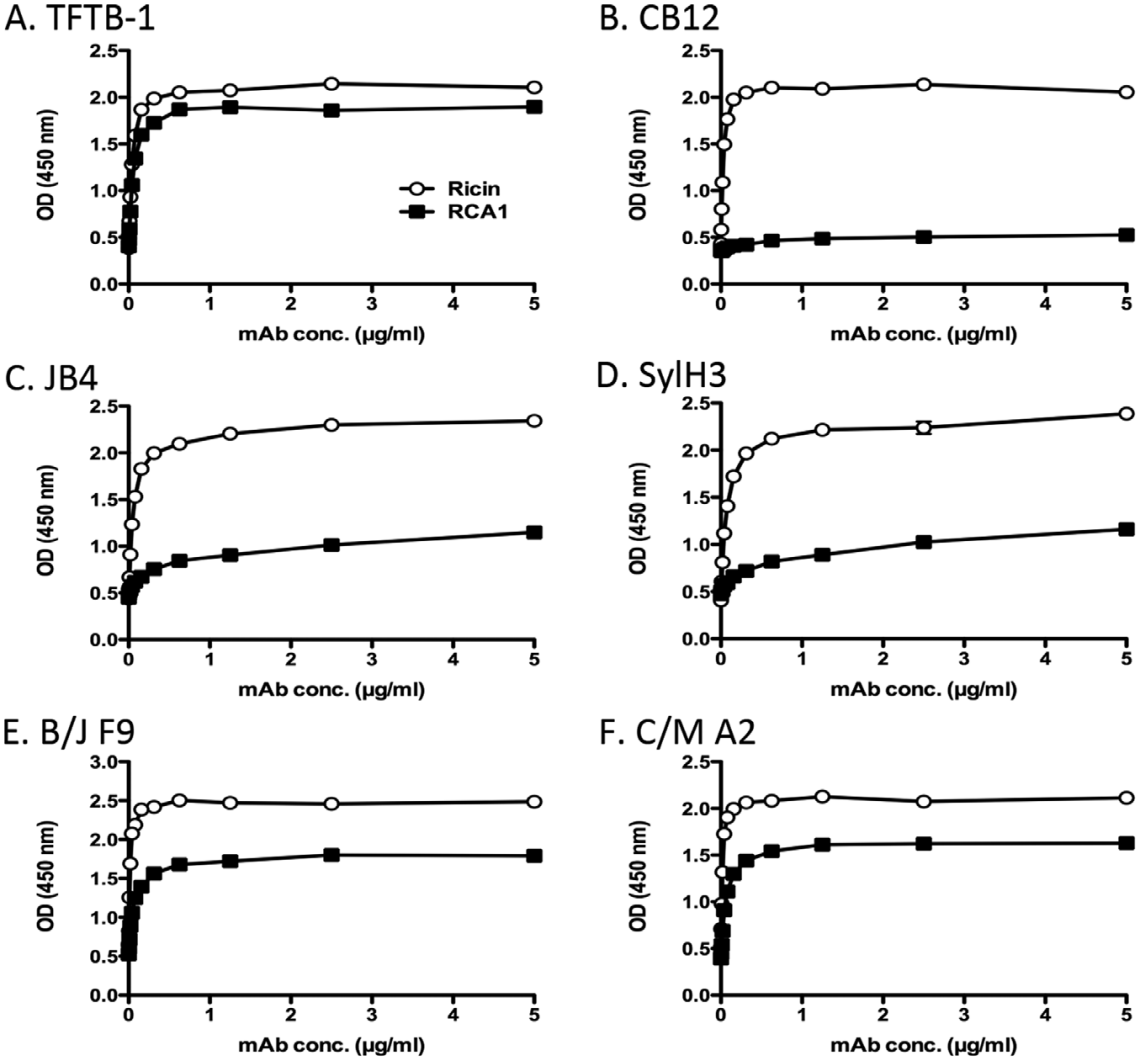
Differential reactivity of RTB-specific mAbs with ricin and RCA-I. (Panels **A-F**) Ninety-six well microtiter plates were coated with 1 μg/ml ricin (open symbols) or RCA-I (closed symbols) and probed with indicated mAbs at the concentrations shown on the x-axis, as described in Materials and Methods. This data is representative of at least 2 independent experiments.

As an additional strategy to localize the epitopes on RTB recognized by JB4, B/J F9, and C/M A2, we examined by ELISA the binding of each mAb to the lectin *Ricinus communis* agglutinin I (RCA-I). RCA-I is a tetrameric glycoprotein consisting of two ricin-like heterodimers whose B subunit (RCB) shares 84% sequence identity with RTB [42], [43]. The utility of RCA-I as a tool in epitope discrimination is exemplified by the differential reactivities of two previously described mAbs, TFTB-1 and CB12 [32]. As shown in **Figure 6 A,B**, TFTB-1 binds equally well to RTB and the RCA-I B subunit (RCB), whereas CB12 recognizes RTB but not RCB. This result is consistent with the fact that TFTB’s epitope is completely conserved between RTB and RCB, whereas only 9/14 residues within CB12’s epitope are present on RCB (**Fig. 6G**). Examination of JB4, B/J F9, and C/M A2 by ELISA revealed that all three mAbs bound RCB to some degree, but considerably less well than they bound RTB (**Fig. 5C-F**). In fact, JB4 (and SylH3) were particularly inept at binding RCB. While these data alone are not sufficient to enable us to pinpoint the exact epitopes recognized by JB4, B/J F9, and C/M A2, they do suggest that the mAbs each bind to a region of RTB that is not fully conserved with RCB (**Fig. 6**).

**Figure 7:**
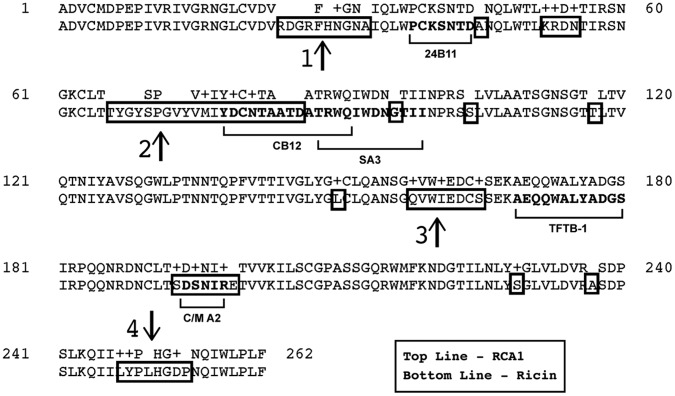
Known and proposed epitopes on RTB recognized by neutralizing and non-neutralizing RTB-specific mAbs. Alignment of the B chain sequences from ricin (PDB 2AAI) and RCA-I (PDB 1RZO) using BLAST (www.ncbi.nlm.nih.gov). Boxed areas indicate regions of differences between RTB and RCB. Bolded text (highlighted by underlying brackets) delineates identified epitopes recognized by RTB-specific mAbs. The name of the corresponding mAb that binds the indicated epitope is written below the brackets. Regions 1-4, as indicated by vertical arrows, reflect regions of difference between RTB and RCB where mAb binding sites have yet to be ascribed.

To further differentiate the mAbs, we examined reactivity by Western blot analysis under conditions in which RTB was (i) solubilized in Laemmli sample buffer, (ii) solubilized in Laemmli sample buffer and boiled (“denatured”), and (iii) solubilized in Laemmli sample buffer with BME and boiled (“denatured and reduced”). We rationalized that the failure of a mAb to bind RTB in its reduced form would indicate that the mAb recognizes an epitope that is constrained by one (or more) of RTB’s four intramolecular disulfide bonds spanning residues C_20_–C_39_, C_63_–C_80_, C_151_–C_164_, C_190_–C_207_. As shown in **Fig. 7A**, C/M A2 bound equally well to RTB in its native, denatured, and denatured/reduced forms, suggesting that C/M A2 binds a linear (continuous) epitope on RTB. Recognition of RTB by B/J F9, on the other hand, was greatly diminished when RTB’s disulfide bonds were reduced, suggesting B/J F9, like 24B11, binds a conformation-dependent (discontinuous) epitope (**Fig. 7B,C**). JB4 recognized all three forms of RTB, although binding was considerably diminished when RTB was reduced (**Fig. 7D**).

### Refined Epitope Mapping using Pepscan Analysis and Phage-displayed Peptide Library

**Figure 8:**
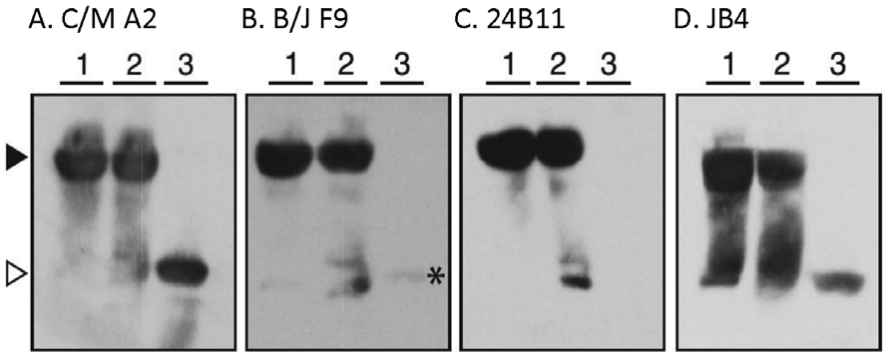
Differential reactivity of RTB-specific mAbs with ricin by Western blot analysis. Ricin holotoxin was suspended in Laemmli sample buffer (lanes 1), suspended in Laemmli sample buffer and boiled (lanes 2), or suspended in Laemmli sample buffer containing β-mercaptoethanol and boiled (lanes 3) before being subjected to SDS-12% PAGE and Western blotting with the indicated mAbs. Panels correspond to the following mAbs: (**A**) C/M A2, (**B**) B/J F9, (**C**) 24B11 and (**D**) JB4. The arrowheads (far left) indicate the location of ricin holotoxin under non-reducing conditions (solid) and RTB (open). Each blot is representative of at least 4 independent experiments.

The fact that C/M A2 bound RTB in its reduced form (see Fig. 6A) suggested that this mAb binds a linear epitope. To localize this epitope, C/M A2 was subjected to pepscan analysis using a collection of 15-mer peptides that overlap by 8 amino acids and span the full length of RTB [32]. While neither JB4 nor B/J F9 reacted with any peptide in the array (data not shown), C/M A2 reacted with two peptides; A8 and C4 (**Fig. 8A**). A8 corresponds to residues T50-L64 (TLKRDNTIRSNGKCL) spanning sub-domains 1β and 1γ, while peptide C4 corresponds to residues C190-I204 (CLTSDSNIRETVVKI) in sub-domain 2β. To determine whether C/M A2 binds to one peptide (“epitope”) preferentially over the other, we performed a competition ELISA in which C/M A2 was pre-incubated with either C4 or A8 peptides at various concentrations before being applied to immobilized ricin in a 96 well plate. As shown in **Fig. 8B**, peptide C4 inhibited the binding of C/M A2 to immobilized ricin in a dose-dependent manner, whereas peptide A8 peptide did not. Peptide C4 was also able to block C/M A2’s ricin neutralization activity in a Vero cell cytotoxicity assay (**Fig. 8C**). Based on these data, we conclude that C/M A2’s epitope likely constitutes residues C190-I204 in sub-domain 2β.

As an alternative strategy to epitope identification, we subjected a phage displayed peptide library to affinity-enrichment using C/M A2 as “bait”, using a protocol recently established in our laboratory (Vance and Mantis, manuscript submitted). After three rounds of panning, 20 phages were isolated by serial dilution on agar plates. Eighteen of the 20 phages bound specifically to C/M A2 by ELISA (**Fig. S1**). Analysis of the peptides encoded by these phages revealed 13 unique sequences that shared a common DxNxR motif (**Fig. 8D**). The DxNxR motif is present in the C4 peptide (CLTSDSNIRETVVKI) and constitutes residues D_194_, N_196_ and R_198_ of RTB. Moreover, there was hydrophobic residue between N and R in every phage we sequenced, which likely reveals the importance of the hydrophobic I_197_ between N_196_ and R_198_ in RTB. There was no universal consensus sequence at the position corresponding to residue 195, although 10/13 phage analyzed contained contain a polar or charged residue in that position similar to residue S_195_.

We also subjected the phage displayed peptide library to affinity-enrichment using B/J F9 as bait, on the off chance that its epitope, although likely discontinuous in nature, could be reconstituted by surface display. We isolated 24 phages after 3 rounds of panning, 20 of which demonstrated specificity for B/J F9. Sequence analysis revealed 5 different peptide sequences that shared a considerable degree of similarity **(Fig. S2)**. Of note, all 5 sequences contained at least two tryptophans, and three of them had 3 tryptophan residues. This is unusual considering that the observed frequency of tryptophan in the phage library is <2% of the random codons. Interestingly, there are only 3 tryptophans exposed on the surface of RTB (W37, W93,W160) and all three are situated in a region that is different from RCB.

**Figure 9:**
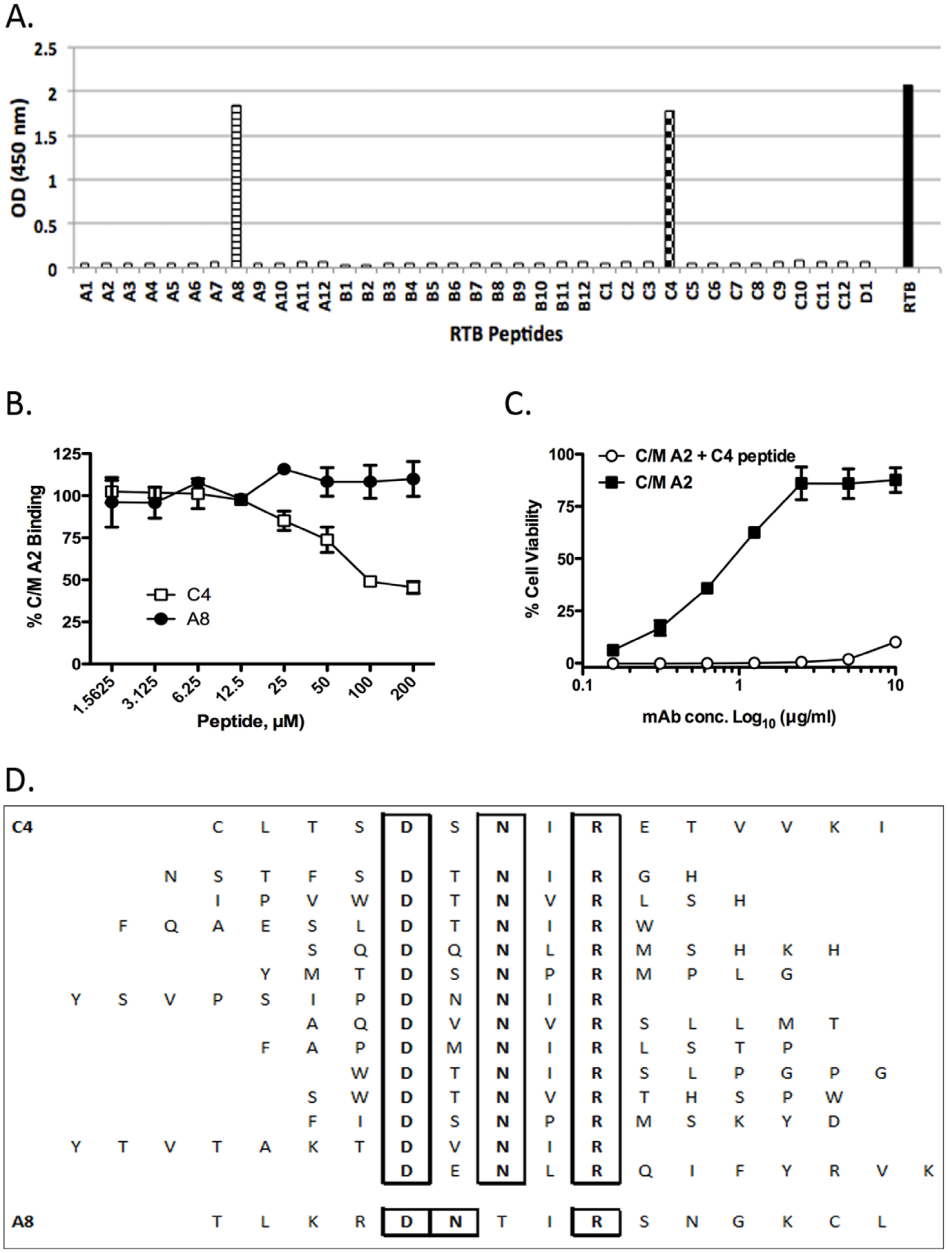
Delineation of the C/M A2 epitope using pepscan analysis and phage display. (**A**) C/M A2 (10 μg/ml: 67 nM) was examined by ELISA for the ability to bind to an RTB peptide array consisting of 37 15-mers (A1-D1, *x*-axis), each overlapping its neighbors by 8 amino acids 32]. C/M A2 reacted with peptides C4 and A8, corresponding to residues T50-L64 (TLKRDNTIRSNGKCL) spanning sub-domains 1β and 1γ and residues C190-I204 (CLTSDSNIRETVVKI) in sub-domain 2β, respectively. Data is representative of 2 independent experiments. (**B**) A competition ELISA in which 96-well microtiter plate coated with RTB was probed with C/M A2 in the presence of increasing concentrations of peptides C4 and A8. (**C**) A competition cytotoxicity assay in which ricin (10 ng/ml) was incubated for 1 hr with C/M A2 mAb at the indicated concentrations in the presence or absence of soluble C4 peptide (10 mM) before being applied in triplicate to Vero cells grown in 96-well microtiter plates. Cell viability was assessed 48 hr later. Data is representative of 2 independent experiments. (**D**) A phage displayed 12-mer peptide library was subjected to affinity enrichment against immobilized C/M A2 mAb, as described in Materials and Methods. The 13 unique sequences identified following 3 rounds of C/M A2 selection are aligned vertically and compared to peptides C4 (top) and A8 (bottom). All 13 phage-derived sequences contained a DxNxR motif (as well as a hydrophobic residue in the 4^th^ position) that aligned exactly with the C4 peptide.

**Figure 10:**
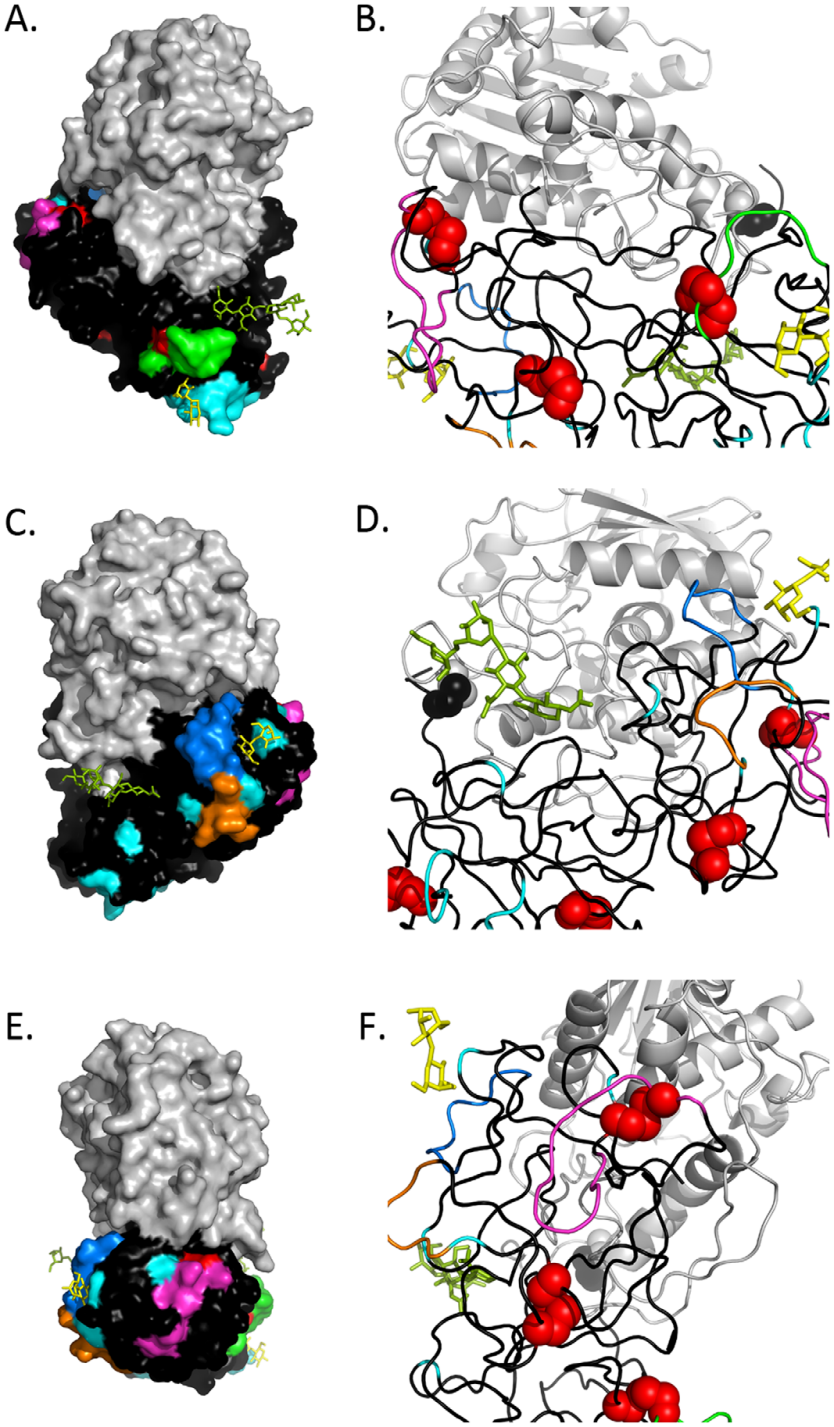
Modeling of proposed B cell epitopes on RTB. We used PyMOL and PBD accession AAI2 to model using surface (left, panels A,C,E) and secondary structure (right, panels B,D,F) depictions of the epitopes recognized by indicated mAbs. Panels A and B: depiction of the epitope recognized by 24B11 (green); panels C and D; depiction of the epitopes recognized by C/M A2 (orange) and JB4/SylH3 (marine blue); panels E and F the epitopes recognized by B/J F9 (magenta). Highlighting common to all panels include RTA (grey), RTB (black), regions of amino acid differences between RCB and RTB (cyan), disulfide bonds (red), mannose side chains (olive green), and lactose (yellow) situated within CRDs.

## Discussion

RTB has two essential roles in ricin cytotoxicity: it mediates toxin attachment to glycoprotein and glycolipids on cell surfaces, and it facilitates the entry and retrograde transport of RTA from the plasma membrane to the ER. Sub-domains 1α and 2γ mediate attachment events; the specific regions of RTB involved in trafficking have yet to be identified. Because RTB is a relatively small protein (34 kDa), we have previously postulated that its association with virtually any antibody (150 kDa for IgG) would have a profound effect on ricin’s ability to bind to host cell receptors and/or engage host cell proteins associated with retrograde transport [44]. Surprisingly, this appears not to be the case. The majority of RTB-specific mAbs that have been produced and characterized to date are in fact non-neutralizing or weakly neutralizing *in vitro* and *in vivo*, despite their high affinities for ricin holotoxin [15], [16], [23], [30], [32]. Moreover, we have estimated in mice that RTB-specific, ricin-neutralizing antibodies constitute only a small fraction of the antibody pool elicited in response to ricin holotoxin or RTB immunization [32]. The results of the current study represent a continuation of our ongoing effort to generate a comprehensive B cell epitope map of RTB and to identify the regions of the protein responsible for eliciting protective antibody responses.

JB4 is only one of a handful of RTB-specific mAbs that have been shown to be capable of conferring passive immunity to ricin in a mouse model [16], [30], [32], [41]. While the exact epitope recognized by JB4 remains unknown, we speculate that it is localized within RTB’s sub-domain 2γ, possibly even encompassing residues 247–254 (**Figs. 6G** **; 9**). JB4 is an extremely potent inhibitor of ricin binding to galactosides, which strongly argues that it recognizes a region of RTB that is in close proximity to one of RTB’s two CRDs. The binding of JB4 to ricin was not affected by 24B11, a mAb that binds an epitope immediately adjacent to the CRD in sub-domain 1α (**Figs. 1** **,** **6G** **; 9 A,B**; [25]). Finally, JB4 reacted poorly with RCB by ELISA, suggesting that JB4’s epitope is not conserved between the two very closely related *Ricinus communis* proteins. The only notable region of difference between RTB and RCB within sub-domain 2γ corresponds to residues 247–254 [25], [32]. On the other hand, we cannot exclude the possibility that even a single amino acid difference at sites other than sub-domain 2γ may account for the differential capacity of JB4 to bind RTB and RCB. Further studies will be required to definitively localize JB4’s epitope on RTB.

It is interesting to note that JB4 recognizes an epitope that is similar if not identical to that recognized by the recently characterized mAb SylH3 [32]. Competition assays by ELISA and SPR revealed that the two mAbs almost completely inhibit one another from binding to ricin (**Table S2**). The two mAbs are not, however, identical, as they were isolated from two independent hybridoma fusions and their V_H_ and V_L_ sequences are different (M. Pauley, personal communication). Moreover, JB4 possesses a slightly higher affinity for ricin than does SylH3 (Table 1). Nonetheless, we think it is significant that JB4 and SylH3, the only protective mAbs we identified from a combined screen of ∼4000 RTB-specific hybridomas, bind the same or a similar epitope. While this result may be fortuitous, we think it more likely that there are a very limited number of neutralizing “hotspots” on RTB and that the epitope(s) recognized by JB4 and SylH3 constitute one of the more immunodominant.

Antibody C/M A2 was originally identified based on its ability to partially neutralize ricin in a Vero cell cytotoxicity assay. Further analysis revealed however, that C/M A2 was unable to inactive ricin in a THP-1 based apoptosis assay, nor was it able to confer any measurable protection against ricin challenge in a mouse model. Although the reason for the difference in C/M A2 neutralizing activities remains unclear, we speculate that it could be due to the MR. In THP-1 cells ricin uptake occurs through two pathways: galactose-dependent, RTB-mediated endocytosis and mannose-dependent, MR-mediated endocytosis [40], [45]. In Vero cells, ricin uptake occurs solely through galactose-dependent, RTB-mediated endocytosis. The fact that C/M A2 neutralizes ricin in a Vero cell assay, but not a THP-1 cell assay, suggests it blocks galactose-dependent, RTB-mediated, but not mannose-dependent MR-mediated uptake of ricin into cells. How this relates to ricin toxicity *in vivo* remains unclear, because we recently reported that mice lacking the MR are more sensitive to ricin that their wild type counterparts [40]. This finding is consistent with the MR playing a role in scavenging ricin from circulation, and not serving as an alternative route of entry into host cells. Further studies are required to fully resolve the importance of the MR, and macrophages in general, in the uptake of ricin into cells *in vivo*.

The observation that C/M A2 recognizes RTB in its reduced and denatured forms enabled us to use two parallel approaches, pepscan analysis and phage displayed peptide library, to define C/M A2’s epitope in great detail. These two approaches, as well as differential reactivity with RCB, strongly implicate residues C190-I204 within sub-domain 2β as being C/M A2’s target on RTB. If correct, then C/M A2 would be the first mAb known to bind RTB’s sub-domain 2β. Interestingly, when modeled by PyMol on the surface of ricin, C/M A2’s epitope is relatively close to the CRD in sub-domain 2β (Fig. 9). However, this distance is apparently too far to occlude the CRD, as evidenced by the failure of C/M A2 to block RTB-galactoside binding.

B/J F9 was also initially selected based on its relatively potent ricin neutralizing activity in the Vero cell cytotoxicity assay. It was therefore quite surprising that B/J F9 failed to confer any passive immunity to ricin in a mouse model. This disconnect likely reflects the shortcomings associated with relying on Vero cells as a primary measure of ricin cytotoxicity, as discussed above. Unfortunately, we were unsuccessful in pinpointing B/J F9’s epitope, although several lines of evidence suggest it localizes within one of two regions; sub-domain 1β (indicted by arrow 2, Fig. 6G) or sub-domain 2α (indicated by arrow 3, Fig. 6G). This conclusion is based on the differential binding of B/J F9 with RCB and RTB by ELISA, limited reactivity of B/J F9 with RTB in its reduced and denatured forms by Western blot, and finally, peptide sequences obtained from affinity enrichment of phage displayed peptide library. The peptides displayed by phage that were capable of binding B/J F9 were enriched in tryptophan residues (**Fig S2**). Sub-domain 1β and sub-domain 2α each contain surface displayed tryptophan, as well as loops formed by an intra-molecular disulfide bonds. Considering the differential binding of B/J F9 to RCB and ricin was not drastically different, we propose that B/J F9 binds sub-domain 2α rather than 1β, as the sequence similarity between RTB and RCB is greater in sub-domain 2α.

We used PyMOL to model the mAb epitopes in 3D. **Fig. 9** shows the epitopes of the mAbs as surface and secondary structures; (**9 A, B**) - previously characterized mAb 24B11, (**9 C,D**) C/M A2 and putative epitope of JB4, and, (**9 E,F**) the putative loop of B/J F9. If B/J F9 binds the proposed loop, then its epitope is located in sub-domain 2α, spatially even further away from the galactose binding site than C/M A2 (**Movies S1 and S2**) and in the same sub-domain as a previously characterized non-neutralizing mAb TFTB-1 [32].

In summary, characterization of three novel anti-RTB mAbs has enabled us to refine our previously constructed B cell epitope map of RTB (Movies S1 and S2). On the whole, our data are consistent with a model in which neutralizing Abs target sub-domains 1α and 2γ, and non-neutralizing Abs target sub-domains 1β, 2α, and 2β. We have not yet identified any mAbs that bind epitopes within sub-domain 1γ. However, based on the fact that it lies between the two other non-neutralizing sub-domains (1β and 2α), and is not involved in galactose binding per se, we think it unlikely that 1γ is a target for neutralizing Abs. The results of this study not only advance our understanding of immunity to ricin, but have important implications for the rational design of RTB-based subunit vaccines. We are currently attempting to express individual sub-domains in RTB with the assumption that sub-domains 1α and 2γ are likely highly efficient at eliciting neutralizing Abs, whereas 1β, 1γ, 2α, and 2β are not. These same constructs will be used to generate additional sub-domain specific mAbs as a strategy to further establish a B cell epitope map of RTB.

## Supporting Information

Figure S1 **C/M A2-specific binding of phage clones.** ELISA showing the ability of representative phage clones to bind to C/M A2 (gray bars), irrelevant IgG Ab MOPC (black bars), RTB (striped bars) or BSA (white bars). Three of these phages bound strongly and specifically to C/M A2, whereas the fourth bound only weakly to C/M A2, and also partially recognized RTB. Of the twenty overall clones isolated, eighteen bound specifically to C/M A2, and all eighteen displayed the DxNxR motif. Correspondingly, the two phages that did not recognize C/M A2 specifically, did not contain the DxNxR motif.
(TIF)

Figure S2 **B/J F9 specific peptide sequences determined using phage display.** Phage display was carried out against mAb B/J F9, and DNA from 24 clones was isolated and sequenced. Of those, 8 unique sequences were shown by ELISA to bind strongly and specifically to B/J F9 (data not shown). Five of these sequences show significant homology, each containing a WxWxP motif (bolded), as well as other conserved residue types (italicized). Two additional peptides also showed partial homology with the motif, while an eighth peptide had no significant homology. Interestingly, all eight peptides have at least one tryptophan residue, and several have multiple tryptophans. This proves the importance of tryptophan in B/J F9 recognition of RTB, as the presence of tryptophan in the random peptide library is expected to be much lower.
(TIF)

Table S1 **List of ricin-specific mAbs.**
(DOCX)

Table S2 **JB4 vs. SylH3 Competition Assays by SPR.**
(DOCX)

Movie S1 **3D rotation of new B cell epitopes on secondary structure of RTB.** PyMOL modeling of ricin, with mAb (putative) epitopes and regions of difference between RTB and RCB highlighted. 24B11 (green), C/M A2 (orange), JB4/SylH3 (marine blue), B/J F9 (magenta), RTA (wheat), RTB (grey), regions of amino acid sequence difference between RCB and RTB (cyane), disulfide bonds (red), mannose side chains (olive green), lactose (yellow).
(MOV)

Movie S2 **3D rotation of new B cell epitopes on surface structure of RTB.** PyMOL modeling of ricin, with mAb (putative) epitopes and regions of difference between RTB and RCB highlighted. 24B11 (green), C/M A2 (orange), JB4/SylH3 (marine blue), B/J F9 (magenta), RTA (wheat), RTB (grey), regions of amino acid sequence difference between RCB and RTB (cyan), disulfide bonds (red), mannose side chains (olive green), lactose (yellow).
(MOV)
