# Supplementary figures and images for: Sub-Domains of Ricin’s B Subunit as Targets of Toxin Neutralizing and Non-Neutralizing Monoclonal Antibodies
Source: PLoS One. 2012 Sep 11;7(9):e44317. doi: 10.1371/journal.pone.0044317 (PMC3439471; doi:10.1371/journal.pone.0044317)

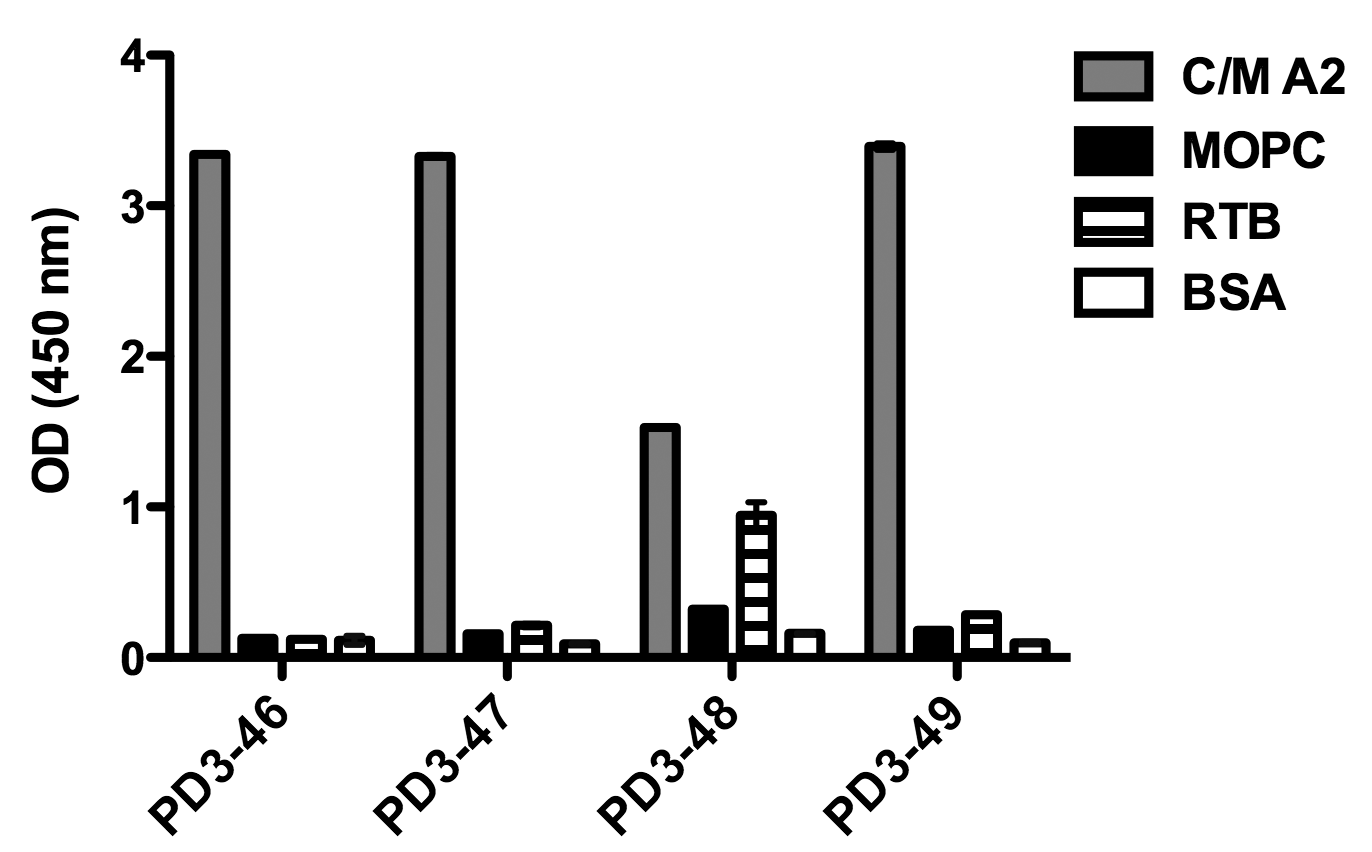

Supplement: Figure S1 — C/M A2-specific binding of phage clones. ELISA showing the ability of representative phage clones to bind to C/M A2 (gray bars), irrelevant IgG Ab MOPC (black bars), RTB (striped bars) or BSA (white bars). Three of these phages bound strongly and specifically to C/M A2, whereas the fourth bound only weakly to C/M A2, and also partially recognized RTB. Of the twenty overall clones isolated, eighteen bound specifically to C/M A2, and all eighteen displayed the DxNxR motif. Correspondingly, the two phages that did not recognize C/M A2 specifically, did not contain the DxNxR motif. (TIF) [file pone.0044317.s001.tif]

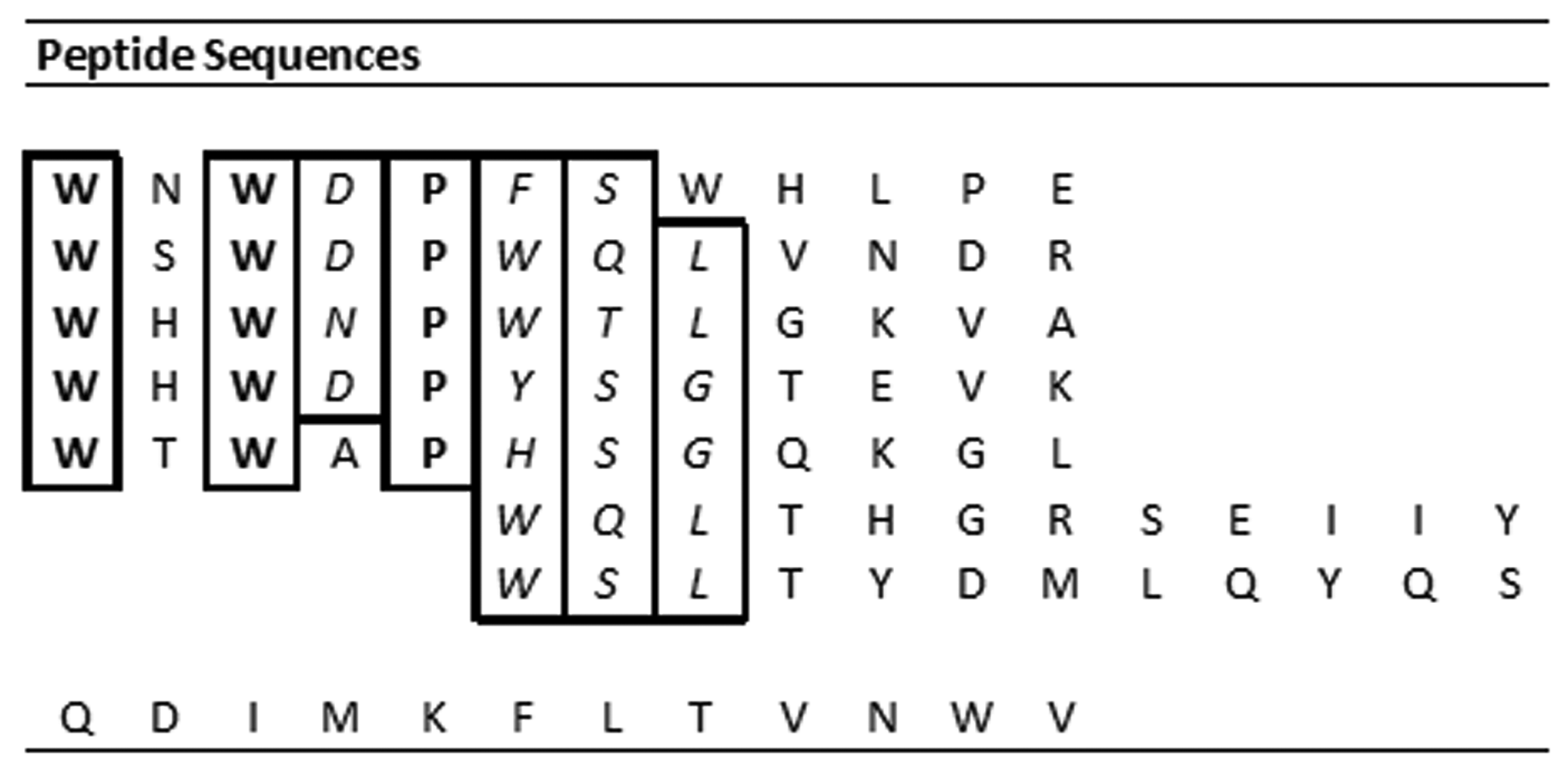

Supplement: Figure S2 — B/J F9 specific peptide sequences determined using phage display. Phage display was carried out against mAb B/J F9, and DNA from 24 clones was isolated and sequenced. Of those, 8 unique sequences were shown by ELISA to bind strongly and specifically to B/J F9 (data not shown). Five of these sequences show significant homology, each containing a WxWxP motif (bolded), as well as other conserved residue types (italicized). Two additional peptides also showed partial homology with the motif, while an eighth peptide had no significant homology. Interestingly, all eight peptides have at least one tryptophan residue, and several have multiple tryptophans. This proves the importance of tryptophan in B/J F9 recognition of RTB, as the presence of tryptophan in the random peptide library is expected to be much lower. (TIF) [file pone.0044317.s002.tif]
