# Supplementary material for: Sub-Domains of Ricin’s B Subunit as Targets of Toxin Neutralizing and Non-Neutralizing Monoclonal Antibodies
Source: PLoS One. 2012 Sep 11;7(9):e44317. doi: 10.1371/journal.pone.0044317 (PMC3439471; doi:10.1371/journal.pone.0044317)
Supplement: Table S1 — List of ricin-specific mAbs. (DOCX) [file pone.0044317.s003.docx]

| **Table S1. List of ricin-specific mAbs** | | |
| --- | --- | --- |
| **Reference** | **Ag***^a^* | **Comments and highlights** |
|  |  |  |
| [[15](#_ENREF_15)] | RT | In vitro characterization of neutralizing and toxin-enhancing RTA- and RTB-specific mAbs |
|  |  |  |
| [[16](#_ENREF_16)] | RTB | Describe mAb 75/3B12; the first demonstration that an RTB-specific mAb is protective in a mouse model |
|  |  |  |
| [[13](#_ENREF_13)] | RT | mAb BG11-G2 confers protection against ricin in a mouse model |
|  |  |  |
| [[22](#_ENREF_22)] | RT | Identified UNIVAX 70 (aka R70) |
|  |  |  |
| [[14](#_ENREF_14)] |  | Anti-idiotype BG11-G2 Abs protect mice against ricin |
|  |  |  |
| [[19](#_ENREF_19)] | ricin | mAbs identified by selection for hybridomas resistant to ricin |
|  |  |  |
| [[23](#_ENREF_23)] | RT, RTA, RTB | Described neutralizing, non-neutralizing, and enhancing RTA- and RTB-specific mAbs |
|  |  |  |
| [[18](#_ENREF_18)] | RT, RTA, RTB | In vitro characterization of RTA-and RTB-specific mAbs; examined mAbs for diagnostic use |
|  |  |  |
| [[20](#_ENREF_20)] | RTB | RTB specific mAb 3E1 that neutralizes ricin |
|  |  |  |
| [[24](#_ENREF_24)] | RT | Production and in vitro characterization of IgA mAbs against RTA and RTB |
|  |  |  |
| [[25](#_ENREF_25)] | RT | Identified an epitope on RTB recognized by a neutralizing mAb |
|  |  |  |
| [[21](#_ENREF_21)] | ricin | RTA and RTB neutralizing mAbs |
|  |  |  |
| [[31](#_ENREF_31)] | n.a. | Ricin specific human-mouse chimeric mAb |
|  |  |  |
| [[26](#_ENREF_26)] | RT | Demonstrated that an RTA-specific IgG mAb confers systemic and intestinal immunity to ricin |
|  |  |  |
| [[28](#_ENREF_28)] | RTA | High affinity scFV from *Macaca fascicularis* |
|  |  |  |
| [[29](#_ENREF_29)] | ricin | Ricin mAbs for diagnostics |
|  |  |  |
| [[27](#_ENREF_27)] | RT | neutralizing and non-neutralizing RTA-specific mAbs |
|  |  |  |
| [[17](#_ENREF_17)] | RTA | Produced and characterized in vitro and in vivo RTA-specific mAbs similar to R70 |
|  |  |  |
| [[30](#_ENREF_30)] | RTA, RTB | Produced neutralizing RTA- and RTB-specific mAbs; |
|  |  |  |
| [[32](#_ENREF_32)] | RT | RTB-specific neutralizing and non-neutralizing mAbs; |

*^a^*Antigen(s) used to generate indicated mAbs.
