# Supplementary material for: Sub-Domains of Ricin’s B Subunit as Targets of Toxin Neutralizing and Non-Neutralizing Monoclonal Antibodies
Source: PLoS One. 2012 Sep 11;7(9):e44317. doi: 10.1371/journal.pone.0044317 (PMC3439471; doi:10.1371/journal.pone.0044317)
Supplement: Table S2 — JB4 vs. SylH3 Competition Assays by SPR. (DOCX) [file pone.0044317.s004.docx]

**Table S2. JB4 vs. SylH3 Competition Assays by SPR.**

| **1^st^ mAb** | **2^nd^ mAb (% Binding)** | |
| --- | --- | --- |
|  | **SylH3** | **JB4** |
| **JB4** | 0.5 | * |
